# Supplementary material for: Accurate image-based identification of macroinvertebrate specimens using deep learning—How much training data is needed?
Source: PeerJ. 2022 Aug 23;10:e13837. doi: 10.7717/peerj.13837 (PMC9415355; doi:10.7717/peerj.13837)
Supplement: Supplemental Information 1 — Figure S1 presents overall variation in accuracy from the cross-validation experiment and Figure S2 presents taxon specific precision and recall from the cross-validation experiment. [file peerj-10-13837-s001.docx]

**Supplementary material**

**Figure S1**

Accuracy in 10-fold cross validation presented separately using majority voting and the max scores sum.


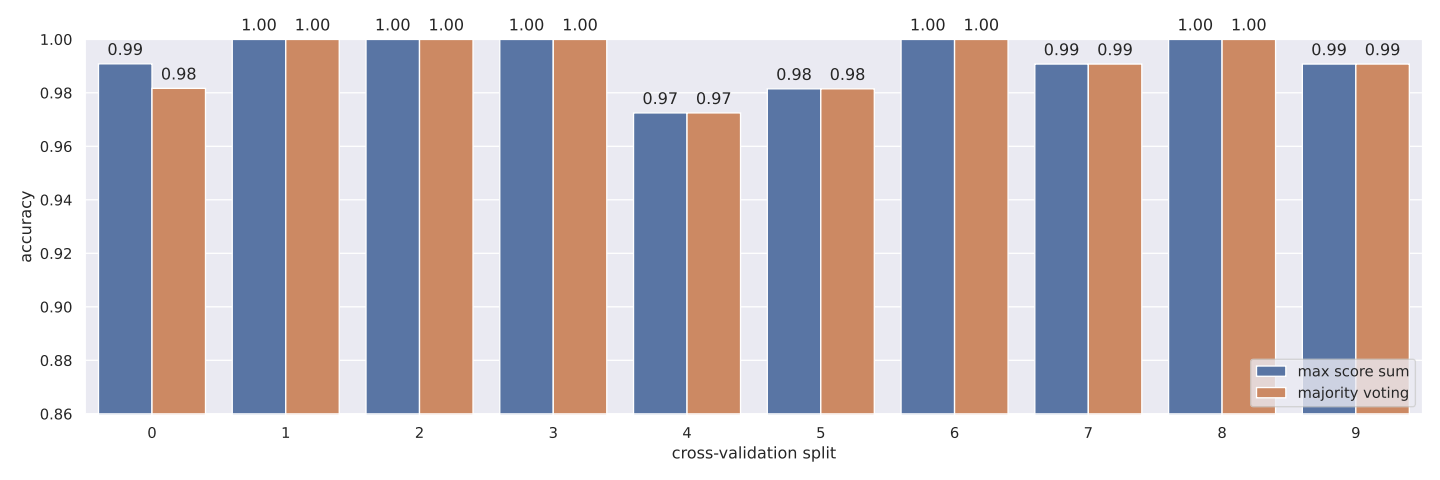


**Figure S2**

Recall and precision from 10-fold cross-validation for each taxon individually.


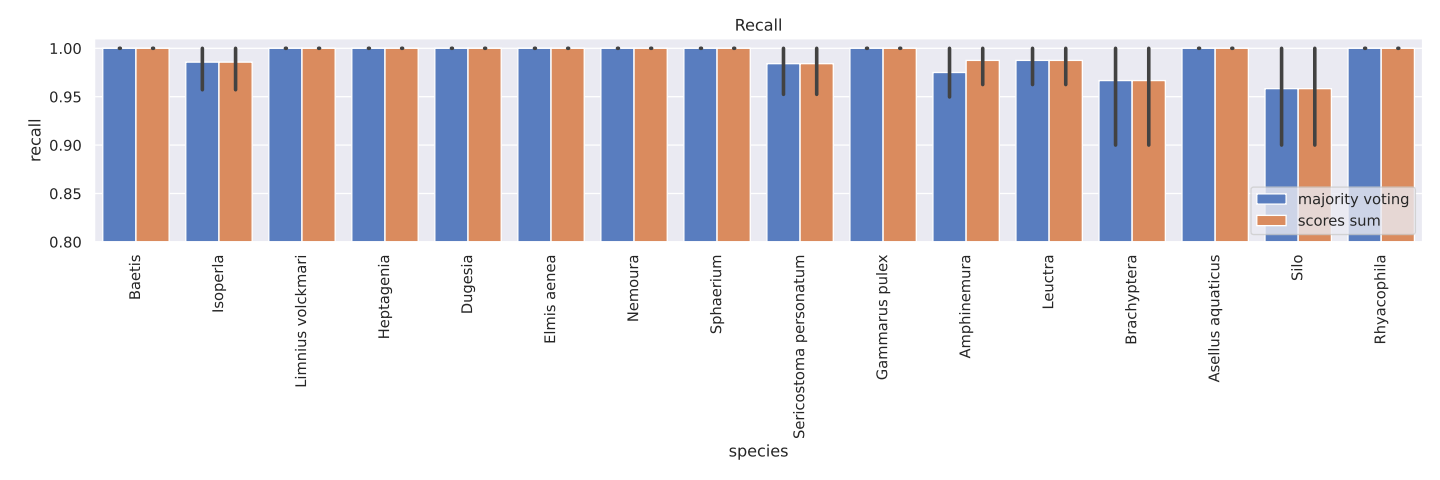


**
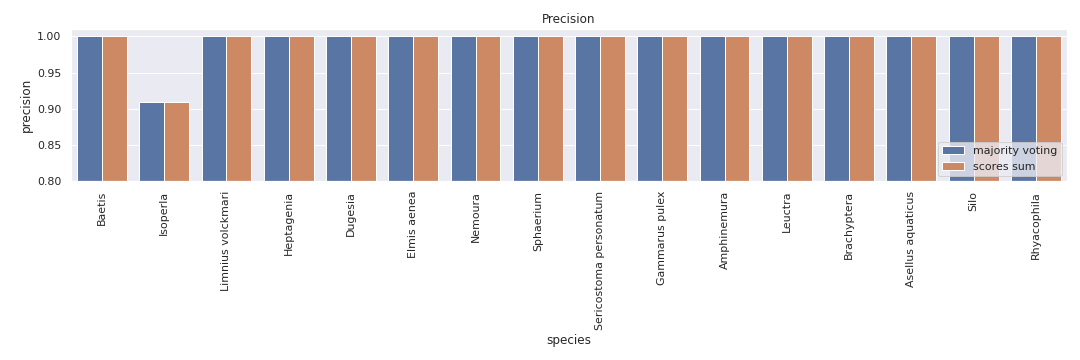
**
